# Influence of Malting Procedure on Volatile Composition of Rye Brome (*Bromus secalinus* L.): A Pilot Study

**DOI:** 10.3390/foods15193382

**Published:** 2026-09-23

**Authors:** Alan Gasiński, Elżbieta Pytlarz, Joanna Śniegowska, Oliwia Hamkało, Inna Mashevska, Magdalena Giemza, Marta Klemens, Telmo Nunes, Joanna Kawa-Rygielska

**Affiliations:** 1 Department of Cereals and Fermentation Technology, Wrocław University of Environmental and Life Sciences, Chełmońskiego 37, 51-630 Wrocław, Poland; 2 Institute of Agroecology and Plant Production, Wrocław University of Environmental and Life Sciences, Grunwaldzki Square 24A, 50-363 Wrocław, Poland; elzbieta.pytlarz@upwr.edu.pl (E.P.);; 3 Department of Genetics, Plant Breeding and Seed Production, Wrocław University of Environmental and Life Sciences, Grunwaldzki Square 24A, 50-363 Wrocław, Poland; inna.mashevska@upwr.edu.pl; 4 DLF Beet Seed ApS, Højbygårdvej 31, 4960 Holeby, Denmark; 5 Department of Food Chemistry and Biocatalysis, Wrocław University of Environmental and Life Sciences, Norwida 25, 50-375 Wrocław, Poland; 6 Gabinete de Apoio Laboratorial—Ciências da Vida, Faculdade de Ciências da Universidade de Lisboa, DBio, Campo Grande, 1749-016 Lisboa, Portugal

**Keywords:** steeping, annual grasses, edible wild plants, herbicide resistance

## Abstract

This study investigated the volatile profile dynamics of wild rye brome (*Bromus secalinus* L.) subjected to a pilot-scale malting run to evaluate its technological potential as a novel grain substitute. Headspace solid-phase microextraction coupled with gas chromatography–mass spectrometry (HS-SPME-GC-MS) was utilized to analyze ground samples of raw seeds (BR-Seed) and malted grain (BR-Malt) in technical triplicates (*n* = 3). A total of 30 volatile compounds were identified across samples (29 in BR-Seed, 24 in BR-Malt). Malting resulted in an overall attenuation of total volatile concentration. Aldehydes constituted the predominant chemical class in both raw seeds and malt, representing 60.13% and 63.00% of total volatilome mass, respectively. Ketones exhibited the most substantial decline during malting, decreasing from 12.50% of total volatiles in seeds to 3.69% in malt, with key off-flavor compounds such as 2-heptanone and 1-octen-3-ol dropping below detection or diminishing substantially. In both seeds and malt, 2-hexenal was present at the highest relative abundance, though its levels decreased 4.35-fold post-kilning (234.53 µg 2-undecanone equivalents to 53.93 µg 2-undecanone equivalents). Crucially, key staling aldehydes (trans-2-nonenal and hexanal) were absent in the malted grain. These pilot-scale baseline findings demonstrate that malting could be an useful method of decreasing various volatiles from rye brome, possibly resulting in a different sensory profile that could support its further development in brewing and baking applications.

## 1. Introduction

Rye brome (*Bromus secalinus* L.), a member of the *Poaceae* family, commonly grows as a weed alongside cultivated cereals [1,2]. Although its grains are not widely utilized in contemporary food technology, historical records indicate that rye brome seeds were occasionally used in food products, mainly due to earlier limitations in separating them from wheat, rye, or oat grains [3]. The presence of rye brome as a weed in cereal crops is increasingly significant because of rising herbicide resistance [1,2].

Advances in grain separation techniques now enable effective removal of rye brome seeds from primary crops such as barley, wheat, or rye [4]. This development opens new opportunities to valorize rye brome grains as a novel raw material, potentially contributing to agricultural waste reduction and diversifying the food product landscape [3]. Moreover, as an annual weed that may re-establish naturally from shed seeds in subsequent seasons, rye brome could serve as an alternative grain source on marginal soils less suitable for conventional cereals, thereby increasing overall grain production [5]. Despite these prospects, the food applications of rye brome grains remain underexplored. Recent investigations into perennial grasses such as intermediate wheatgrass (*Thinopyrum intermedium*) revealed limitations in bread making due to weak protein functionality [6]. This highlights the need to explore alternative uses that do not depend on strong gluten networks. One such approach is malting—a process involving steeping, controlled germination, and kilning—that enhances enzymatic activity and alters the biochemical composition of grains, with significant impact on flavor and aroma compounds. A recent study has shown that rye brome malt with adequate technological properties can be obtained [7]. Malting initiates complex metabolic processes resulting in the formation of volatile molecules that can contribute to the sensory profile of malt-based products [8]. Therefore, there exists a possibility of a study aimed at characterizing its volatile composition and changes in this composition after the malting procedure. The volatile profile of malted grains strongly influences the aroma and flavor attributes of final food and beverage products. To date, no comprehensive analysis of the volatile compounds present in rye brome seeds and rye brome malt has been conducted. Understanding these constituents is essential to evaluate the potential of rye brome malt as a source of unique flavors. The presented study evaluated the volatile profile of rye brome (*Bromus secalinus* L.) subjected to a pilot-scale malting run. Because processing wild or underutilized grain species at an expanded batch volume introduces novel physical dynamics during steeping, germination, and kilning, establishing an initial baseline dataset is essential. This work was designed as an exploratory pilot run evaluated via technical triplicates to descriptively characterize the primary volatile shifts, losses of volatiles, and sensory-active compound formation occurring between raw seeds and malted grain.

## 2. Materials and Methods

### 2.1. Materials

#### 2.1.1. Plant Material

Plant material used in this study consisted of seeds collected from a herbicide-resistant biotype of rye brome (*Bromus secalinus* L.). This biotype exhibited a low level of resistance (R = 2–4) to pyroxsulam. Figure 1 and Figure 2 present the rye brome plants from which the grains used in this study were harvested.

#### 2.1.2. Chemical Reagents

The chemical reagents used in this study were as follows: 2-undecanone (99%); 2-(E)-hexenal (98%); 1-hexanol (99%); 2-heptanone (98%); heptanal (95%); 2-(E)-heptenal (95%); benzaldehyde (99%); 1-heptanol (99.5%); 1-octen-3-ol (98%); octanal (99%); 2-cyclohexen-1-one (95%); 2-ethyl-1-hexanol (99%); phenylacetaldehyde (95%); 2-(E)-octenal (95%), 2,3-octanedione (95%); acetophenone (98%); 3,5-octadien-2-one (95%); 1-octanol (99%); nonanal (95%); 2,6,6-trimethyl-2-cyclohexene-1,4-dione (98%); decanal (98%); 1-decanol (99%); undecanal (99%); valencene (70%). All reagents were purchased from Merck company (Merck, Darmstadt, Germany).

### 2.2. Methods

#### 2.2.1. Malting Procedure

The malting procedure consisted of steeping, germination, and drying. Rye brome seeds with a water content of 7% (*w*/*w*) were loaded into stainless steel perforated containers (50 g of seeds per container) and submerged in water at a water-to-grain ratio of 10:1 (water temperature, 15 °C) for 5 h (a total of 4 containers). After 5 h of steeping with no external aeration, the water content of the seeds increased to 50–52% (*w*/*w*). The containers were then transferred to a KK 240 Smart Plus germination chamber set at 15 °C and 90% relative humidity. Every 24 h during germination, the seeds were sprayed with distilled water (an amount adequate to keep the container weight constant) and mixed. Although a 120 h germination period (standard for conventional barley malt) was initially planned, rapid elongation of the rye brome acrospires and rootlets required shortening the germination phase to 72 h. Following germination, the containers were transferred to a UF110 Plus laboratory dryer (Memmert GmbH + Co., Schwabach, Germany) and dried using the following temperature program: 50 °C (18 h 50 min), increase to 70 °C (10 min), 70 °C (2 h), increase to 80 °C (10 min), and 80 °C (2 h). Upon completion of drying, the rootlets of the rye brome malts were removed, and the resulting malts (5% *w*/*w* moisture content) were mixed and transferred to a polypropylene container. The container was airtightly closed to mitigate moisture absorption by the malt. Malt (BR-Malt) and unmalted seeds (BR-Seed) were then ground using a Bühler Miag disc mill DLFU (Bühler, Uzwil, Switzerland), with the gap between the discs set at 0.2 mm. Both samples, BR-Seed and BR-Malt, were kept in the same type of container, for the same duration before analysis.

#### 2.2.2. Extraction of Volatiles to Solid-Phase Microextraction (SPME) Fiber

A ground sample (2 g of seed or malt) was weighed into a 20 cm^3^ headspace vial. Eight cm^3^ of saturated NaCl solution was added to the vial, followed by the addition of 50 uL of internal standard (IS, 2-undecanone in cyclohexane, 1 mg/1 dm^3^). The vials were placed on the heating plate of a magnetic stirrer, set at 200 rpm and 25 °C. The needle of the SPME holder was inserted through the septum of the vial and the fiber (DVB/CAR/PDMS 50/30 µm; 1 cm length; Merck, Darmstadt, Germany; preconditioned according to the manufacturer suggestions) was introduced into the vial. The sample was equilibrated for 5 min, after which the fiber was exposed to the headspace above the sample. Extraction of the volatiles was performed for 40 min for each sample, in triplicate. A low volatile extraction temperature was used due to the formation of Maillard reaction by-products during the extraction of volatiles at higher temperatures.

#### 2.2.3. Analysis of Volatile Compounds in Rye Brome Seeds and Malts

Volatile profiling of seeds and malts was performed using gas chromatography coupled with mass spectrometry (GC-MS). Analyses were performed using a GC-2010 Plus chromatograph coupled with a GCMS-QP2010 SE mass spectrometer (Shimadzu, Kyoto, Japan). A ZB-5 column (Phenomenex, Torrance, CA, USA) with a 30 m length, 0.25 mm diameter, and 0.25 µm film thickness was used to separate volatiles. Injection port temperature was held at 195 °C. The carrier gas used was helium (99.9999% purity) at a flow rate of 1.78 cm^3^ per min. The pressure at the start of the analysis was set at 100 kPa. The temperature program used throughout each of the analyses was as follows: 40 °C, hold for 1 min, ramp up at a rate of 8 °C per min to 195 °C, hold for 5 min. The temperature of the ion source was maintained at 250 °C, and the interface temperature was at 195 °C. Splitless injection was used, with a desorption time equal to 2 min. Scanning was carried out in the 35–350 m/z range using 70 eV electron ionization, with an event time equal to 0.3 s (scan speed equal to 1111). Retention times and mass spectra of the original compounds, with the exception of 1-(2-methyl-1-cyclopenten-1-yl)-ethanone, were used to confirm the identification of the compounds present in rye brome seeds and rye brome malts. Linear retention indices and the NIST spectral library were used for the identification of 1-(2-methyl-1-cyclopenten-1-yl)-ethanone (similarity search was equal to 95%); 2,6,6-rimethyl-2-cyclohexene-1,4-dione (similarity search equal to 93%); 2-(2-butoxyethoxy)-ethanol (similarity search equal to 91%); 2-phenoxy-ethanol (similarity search equal to 94%); 2-(2-butoxyethoxy)-ethanol (similarity search equal to 91%); dihydro-5-pentyl-2(3H)-furanone (similarity search equal to 95%); (-)-α-Panasinsen (similarity search equal to 93%); and epi-γ-Eudesmol (similarity search equal to 95%). The C6-C20 alkane mixture in hexane was used to acquire retention indices [9]. Quantification was conducted on a semi-quantitative basis relative to the internal standard 2-undecanone (50 ng added per sample). The relative concentration of each identified volatile was calculated by dividing its chromatographic peak area by the peak area of 2-undecanone, multiplying by the mass of the internal standard, and normalizing to the dry sample mass, taking into account the seed/malt moisture content. Because compound-specific relative response factors and HS-SPME matrix extraction efficiencies were not applied, all reported values represent semi-quantitative concentrations expressed as 2-undecanone equivalents (ppb IS equivalents).

#### 2.2.4. SEM Microscopy

A scanning electron microscope was utilized to obtain close-up pictures of rye brome malt grain and husk samples to showcase the size differences between those structures present in this malt. Plant material was initially fixed in 1.5% glutaraldehyde (GA) in 0.05 M sodium cacodylate buffer at pH 7.0 for 45 min at room temperature. Following 1–2 min under a vacuum (26 mm Hg, 3.46 kPa), the initial fixative was replaced with a 3% GA solution in 0.1 M sodium cacodylate buffer at pH 7.0 and incubated for 2 h at room temperature. The material was then thoroughly rinsed with the same buffer and subjected to post-fixation in a 1% osmium tetroxide (OsO_4_) aqueous solution for 2 h at room temperature. Following dehydration in a graded series of acetone, the material was dried in a Polaron E3500 critical point dryer (Polaron Equipment Ltd., Watford, UK). The dried samples were sputter-coated with gold in a Polaron E5350 vacuum evaporator (Polaron Equipment Ltd., Watford, UK), and the observations were made using a Jeol JSM T220 scanning electron microscope (JEOL Ltd. Akishima, Japan) The methodology employed is described thoroughly by Figueiredo & Pais [10].

#### 2.2.5. Data Analysis

All GC-MS volatile profiling measurements were performed in technical triplicates (n = 3) using homogenized samples derived from the pooled pilot scale-up malting run (n = 1). Analytical results are presented descriptively as ‘mean ± standard deviation’ across technical replicate injections to reflect analytical precision and as estimated relative abundances in µg 2-undecanone equivalents per kg dry matter (µg eq./kg DM). Changes in the concentrations of volatiles present in BR-Seed and BR-Malt were calculated as relative baseline fold changes using mean value ratios of the concentration determined in BR-Seed and BR-Malt (or vice versa). Compounds were classified as ‘not detected’ if their chromatographic peak area fell below three times the standard deviation of the baseline noise. To reduce the possibility of compromising the identification of compounds present in the rye brome seeds or rye brome malts, blank runs determining volatiles from the glass liner and septum; SPME fiber; internal standard (the internal standard was injected at a split of 20); and polypropylene containers which were used to store the samples (SPME fiber was inserted into the hole drilled in the cap of one of the containers and exposed to the air present in the container for 60 min at ambient temperature) were run in duplicate, and the peaks present in these analyses were discarded from the group of identified compounds detected in the rye brome malts and grains.

## 3. Results and Discussion

The GC-MS analysis allowed for the identification and quantification of 30 volatile compounds across samples, 29 of which were detected in BR-Seed and 24 in BR-Malt, as presented in Table 1 and Table 2. The identified compounds consisted of aldehydes (nine different compounds identified in BR-Seed, 10 in BR-Malt); alcohols (eight identified in BR-Seed and BR-Malt); ketones (nine in BR-Seed, three in BR-Malt), as well as terpenes and terpenoids (three in BR-Seed and BR-Malt). Figure 3 and Figure 4 illustrate the proportional mass contribution of these respective volatile categories to the overall profile of rye brome seeds and malts (the calculation of this mass contribution is provided in the Supplementary Materials).

Table 1 presents retention indices, reported odor descriptors of the compounds, as well as the method of identification used to determine particular volatiles in the rye brome seeds and malts.

Table 2 presents concentration of all the volatiles identified and quantified in the rye brome seeds and malts.

The volatile compositional profile of rye brome seeds and malts reveals dynamic shifts that depart from standard cereal malting behaviors. In conventional malting, grain steeping and germination trigger extensive metabolic cascades: amylases hydrolyze starch into fermentable sugars, while proteases cleave proteins into free amino acids, modifying the endosperm protein-starch matrix. During kilning, particularly in early, high-moisture stages, reducing sugars and free amino acids undergo Maillard reactions to synthesize diverse volatile complexes [29].

Additionally, during malting, germinating grains exhibit lipolytic, lipoxygenase (LOX), and hydroperoxide lyase (HPL) activities that convert free fatty acids into volatile esters and aldehydes. Minor enzymatic pathways include β-glucosidase cleavage of sugar-bound terpene/phenolic precursors and phenolic oxidation [30].

The predominant compound in both raw seed and malt was 2-hexenal (234.53 µg eq./kg DM in seeds vs. 53.93 µg eq./kg DM in malt), representing a 4.35-fold reduction during malting. In conventional grain processing, 13-fatty acid hydroperoxide lyase (13-HPL) acts on linoleic or α-linolenic acids to form 3(Z)-hexenal, which rapidly isomerizes to 2-hexenal [31]. The baseline levels observed here suggest relatively constrained endogenous enzymatic synthesis in rye brome relative to thermal loss during kilning.

1-Hexanol estimated relative abundance exhibited a 3.56-fold decrease from seeds (57.97 µg eq./kg DM) to malt (16.28 µg eq./kg DM). While yeast synthesizes hexanol via reduction of 2-hexenal and hexanal during fermentation, yeast-driven pathways are absent during malting. The presence of 1-hexanol in raw grain can possibly stem from endogenous seed metabolism or epiphytic microflora on the grain surface, while its decline in malt might be driven by thermal volatilization during kilning [32].

A similar trend occurred for 1-octanol, which decreased 3.90-fold (15.68 µg eq./kg DM to 4.02 µg eq./kg DM). Because, generally, plant 1-octanol originates primarily from oleic acid lipid oxidation, its lower prevalence in malt might suggest either a limited endogenous oleic acid substrate pool or thermal evaporation exceeding the rate of enzymatic generation during drying [33].

2-Heptanone, a ketone that is typically reported to have a cheesy off-flavor and is derived from lipid oxidation, dropped below detection limits in malt (from 13.13 µg eq./kg DM). This complete loss may suggest either a limited endogenous pool of precursor lipids or perhaps a strong protective antioxidant activity in rye brome that limits lipid autoxidation during processing [34].

Heptanal estimated relative abundance in BR-Seed (7.72 µg eq./kg DM) was 3.69 times higher than in BR-Malt (2.09 µg eq./kg DM). Previous research by Li et al. (2020) demonstrated that heptanal can be synthesized through the enzymatic oxidation of both palmitoleic and oleic acids [35]. The lower baseline concentration in malt may suggest that raw rye brome seeds might possess a relatively small pool of these specific fatty acid precursors or exhibit limited enzymatic activity during germination. Consequently, thermal losses during the kilning and drying procedure appear to outpace de novo enzymatic generation. A parallel trend was observed for 2-heptenal, which is typically formed via analogous lipid-oxidation pathways. Its estimated relative concentration decreased 1.68-fold from BR-Seed (6.65 µg eq./kg DM) to 3.95 µg eq./kg DM in BR-Malt, reinforcing the hypothesis that thermal evaporation during drying can predominate over enzymatic synthesis for these specific medium-chain aldehydes in rye brome.

Heptanol semi-quantitative content dropped 6.29-fold (9.88 µg eq./kg DM in BR-Seed vs. 1.57 µg eq./kg DM in BR-Malt). Unlike standard six- or nine-carbon LOX derivatives, 1-heptanol generally forms via a hybrid pathway: non-enzymatic autoxidation of oleic acid to heptanal, followed by alcohol dehydrogenase (ADH) reduction in the germinating embryo [33,36]. The steep decline in malt might point to low endogenous ADH activity relative to the rate of evaporative loss during kilning. 1-Octen-3-ol decreased 2.47-fold (13.57 µg eq./kg DM to 5.49 µg eq./kg DM). Formed either through LOX activity or non-enzymatic autoxidation of linoleic acid, 1-octen-3-ol is associated with reported mushroom-like, earthy off-notes. Although sensory panel testing was not conducted in this study, the decrease in this volatile during malting may alter the aroma profile of downstream food or beverage products [32,37]. Its reduction during malting might represent a potential sensory benefit for beverage and food applications [25].

Other secondary lipid-oxidation products derived from specific unsaturated fatty acids likewise decreased. The estimated relative abundance of 3-Octen-2-one was reduced 3.89-fold (from 7.75 µg eq./kg DM in BR-Seed to 1.99 µg eq./kg DM in BR-Malt). As this compound is also formed via secondary oxidation from the same linoleic acid precursor as 1-octen-3-ol, its greater rate of depletion further reinforces the hypothesis that linoleic-derived pathways are limited or offset by thermal loss [33].

The estimated relative abundance of 2,3-octanedione decreased 3.27-fold in BR-Malt (10.65 µg eq./kg DM to 3.26 µg eq./kg DM). A similar depletion pattern to that of 3-octen-2-one, possibly influenced by thermal autoxidation and photooxidation, was observed, with the key distinction that the primary fatty acid source for 2,3-octanedione is linolenic acid rather than linoleic acid [33].

3,5-Octadien-2-one shares the same linolenic acid origin as 2,3-octanedione, and it decreased 8.51-fold (from 9.70 µg eq./kg DM in BR-Seed to 1.14 µg eq./kg DM in BR-Malt), supporting the hypothesis of a potential baseline decline in linolenic-derived volatiles [33].

Non-linear and cyclic volatile compounds were completely lost during the malting procedure. 2-Cyclohexen-1-one (5.23 µg eq./kg DM in BR-Seed), acetophenone (3.16 µg eq./kg DM), 1-(2-methyl-1-cyclopenten-1-yl)-ethanone (11.30 µg eq./kg DM), and dihydro-5-pentyl-2(3H)-furanone (4.29 µg eq./kg DM) all dropped below the detection limit in BR-Malt.

The loss of 2-cyclohexen-1-one is notable, as current literature on grain germination and roasting primarily details the formation and loss of linear ketones. Available evidence indicates that while linear ketones can actively generate and volatilize during thermal processing, cyclic ketones lack active regeneration pathways during germination and are particularly susceptible to thermal degradation or stripping [38].

Regarding acetophenone, this compound is a documented natural metabolite in plants and surface microflora, whose physicochemical properties facilitate cellular diffusion [39]. The trace levels detected in BR-Seed (3.16 µg eq./kg DM) may have originated from the metabolic activity of epiphytic microbiota on the seed coat, with its complete absence in malt pointing to volatilization during kilning without subsequent regeneration. Similarly, it is possible that 1-(2-methyl-1-cyclopenten-1-yl)-ethanone and dihydro-5-pentyl-2(3H)-furanone evaporated during thermal drying rather than accumulating through active metabolic steps during steeping and germination.

Only three volatile compounds displayed higher relative concentrations in BR-Malt than in BR-Seed. 2-Octenal, absent in BR-Seed, accumulated to 2.30 µg eq./kg DM in BR-Malt. Formed via cleavage of linoleic acid hydroperoxides via lipase and LOX pathways, its accumulation may indicate enzymatic activation during germination [40]. Decanal increased 1.80-fold from 13.07 µg eq./kg DM to 23.52 µg eq./kg DM. Decanal arises primarily through enzymatic conversion of oleic acid, a pathway widely documented across conventional grains (barley, rye, wheat, and oats) [41]. 1-Decanol increased 1.41-fold (3.77 µg eq./kg DM to 5.31 µg eq./kg DM). This accumulation aligns with active enzymatic reduction of its parent aldehyde, decanal, during germination, which could possibly explain its increase [42].

Compounds such as octanal (6.01 vs. 6.05 µg eq./kg DM), nonanal (14.41 vs. 12.72 µg eq./kg DM), undecanal (2.23 vs. 2.31 µg eq./kg DM), and 2-phenoxyethanol (6.90 vs. 5.91 µg eq./kg DM) remained relatively stable. Although linear aldehydes possess high vapor pressures and moderate boiling points (~171–223 °C), there is possibility that ongoing enzymatic formation during germination could offset thermal loss during kilning [43,44].

The endogenous sesquiterpenes, valencene (7.20 to 2.35 µg eq./kg DM, 3.06-fold decrease), (-)-α-panasinsen (9.64 to 3.70 µg eq./kg DM, 2.61-fold decrease), and epi-γ-eudesmol (15.98 to 7.03 µg eq./kg DM, 2.27-fold decrease), showed a uniform decrease. Because sesquiterpenes are specialized plant defense metabolites synthesized via the isoprenoid pathway during vegetative field growth, mature seeds lack de novo synthesis capacity [45]. Their observed decline therefore possibly reflects pure thermal volatilization during the drying stage.

Benzaldehyde and phenylacetaldehyde both exhibited substantial baseline reductions exceeding 22-fold from BR-Seed to BR-Malt. Benzaldehyde dropped from 25.23 µg eq./kg DM to 1.10 µg eq./kg DM (22.94-fold decrease), while phenylacetaldehyde dropped from 25.12 µg eq./kg DM to 1.14 µg eq./kg DM (22.04-fold decrease). Phenylacetaldehyde forms via Strecker degradation of phenylalanine, subsequently oxidizing to benzaldehyde. This large decrease suggests that perhaps free phenylalanine may be preferentially directed toward protein synthesis in developing rootlets rather than accumulating for Strecker pathways or just that rye brome proteins are characterized by a small concentration of phenylalanine [42].

Cramer et al. investigated volatiles in the seeds of various barley and wheat cultivars, while concentrating on differences in the volatilome between hulless and hulled barley seeds [46]. As the rye brome seeds are characterized by a rather large hull proportion relative to the total grain size (as shown by the SEM micrographs of BR-Malt in Figure 5), perhaps some of the results obtained could thus be explained.

Seeds of hulled barley varieties contained more aldehydes and more alcohols than those of the hulless barley varieties. They also contained more fatty acids, which, as mentioned before, could be substrates for the formation of a plethora of identified volatiles. Therefore, there exists a possibility that the large proportion of the husk in rye brome seeds could be responsible for the high concentration of aldehydes and alcohols in BR-Seed as well as BR-Malt.

Compared to barley malt volatile profiles reported by Ma et al. (2024), BR-Malt contained elevated 2-hexenal levels but drastically lower levels of off-flavor markers like 2-heptanone and phenylacetaldehyde [47].

Furthermore, key ‘beer staling’ aldehydes described by Filipowska (specifically trans-2-nonenal and hexanal) were completely absent in BR-Malt, while phenylacetaldehyde levels were 10- to 100-fold lower than in typical barley malts [42].

The volatile composition of grains also plays a part in another significant branch of food production, which is bread and pasta production. Current trends indicate that consumers want to try products created using not only wheat or rye but also other cereals and pseudocereals [48,49].

Similar comparative patterns to those observed between barley malts and BR-Malt emerge in baking applications: semolina flour displays higher heptanal and heptanone levels than rye brome malt, as shown in the study by Bellagia et al. (2009), while the concentrations of octanal, nonanal, and decanal in BR-Seed and BR-Malt were higher than in semolinas [50].

The impact of the hull on the overall volatilome of grain products was also described by Czerny & Schieberle (2002), who analyzed white wheat flour and wholemeal flour [51]. Wholemeal flour contained a vastly higher concentration of phenylacetaldehyde, indicating that this component is primarily localized in the husk. Consequently, this suggests that drying process likely drives the depletion of phenylacetaldehyde from the grain husk.

Overall, these pilot findings demonstrate that malting rye brome can possibly influence the concentration of off-flavor compounds (e.g., 2-heptanone or 1-octen-3-ol) while minimizing staling aldehyde precursors. This overall volatile change might perhaps position rye brome malt as a grain substitute for brewing and novel food formulations.

## 4. Conclusions

Malting rye brome seeds has a different effect on the volatile profile of the sample than is typically observed in other malted cereals. Rye brome seeds, after malting, contain a lower relative abundance of various volatiles than unmalted rye brome seeds. Unlike standard brewing grains, which typically exhibit an accumulation of volatile compounds during processing—including potential off-flavor precursors in food applications such as brewing or baking—rye brome showed a general attenuation across most identified volatile classes following malting. While these baseline shifts in volatile abundance suggest that malting may alter the aroma characteristics of raw rye brome, formal sensory panel evaluations are required to determine the precise perceptual and organoleptic implications for food and beverage applications. The role of malting in the management of herbicide-resistant populations of *B. secalinus* in agricultural systems requires further investigation.

## Figures and Tables

**Figure 1:**
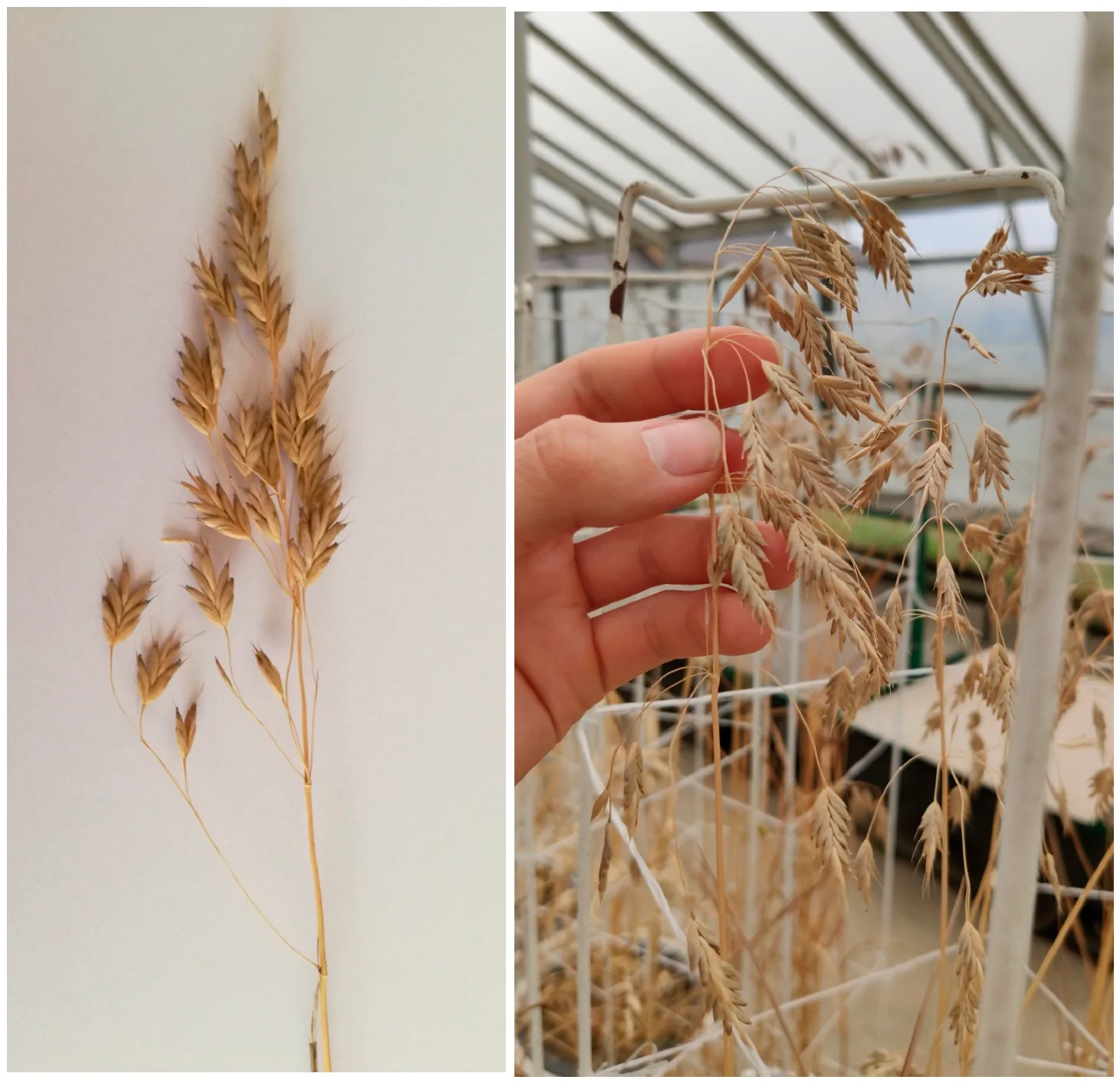
Inflorescences of the rye brome in the maturity phase.

**Figure 2:**
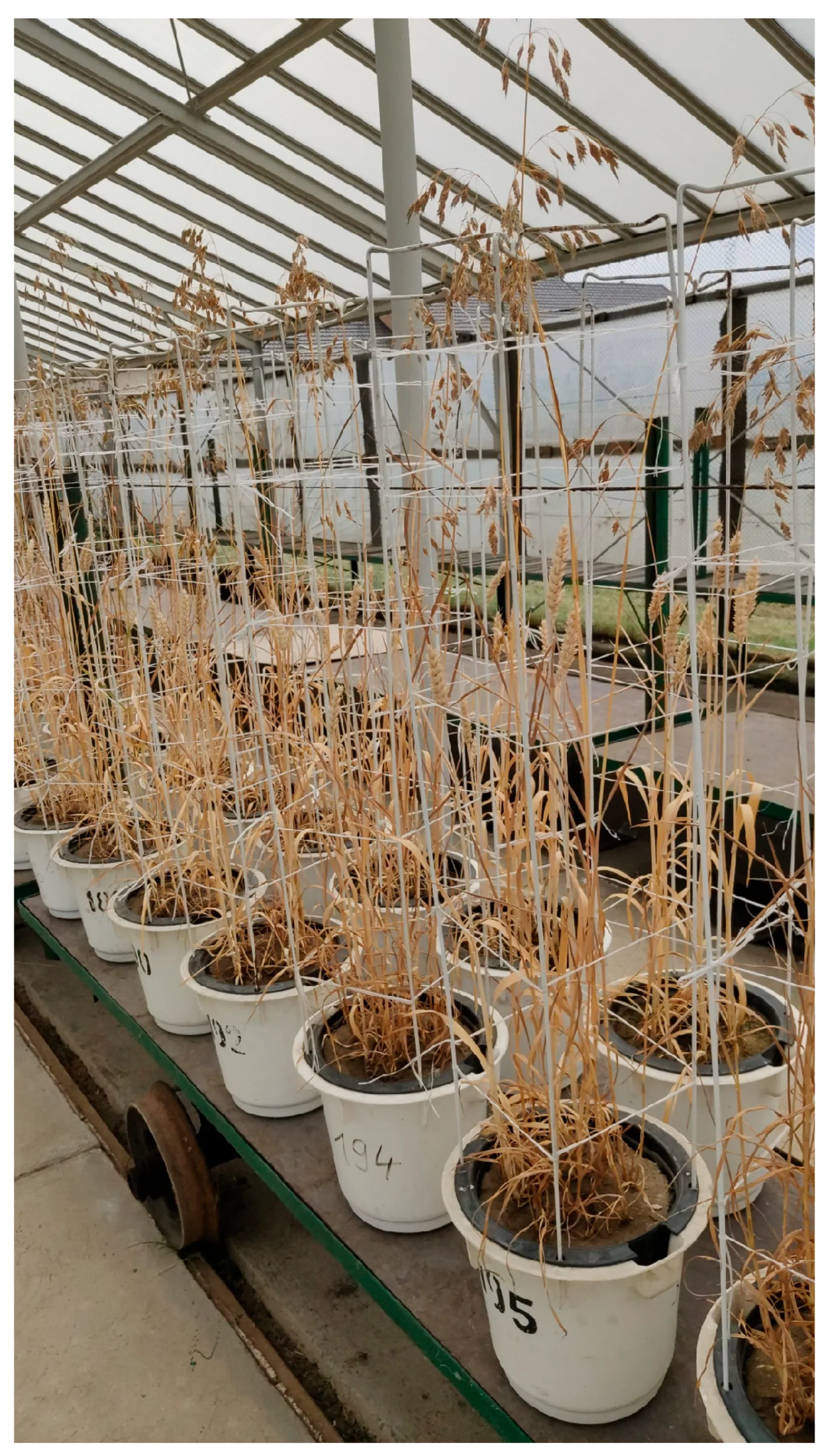
Pot experiment in which the rye brome used in this study was grown.

**Figure 3:**
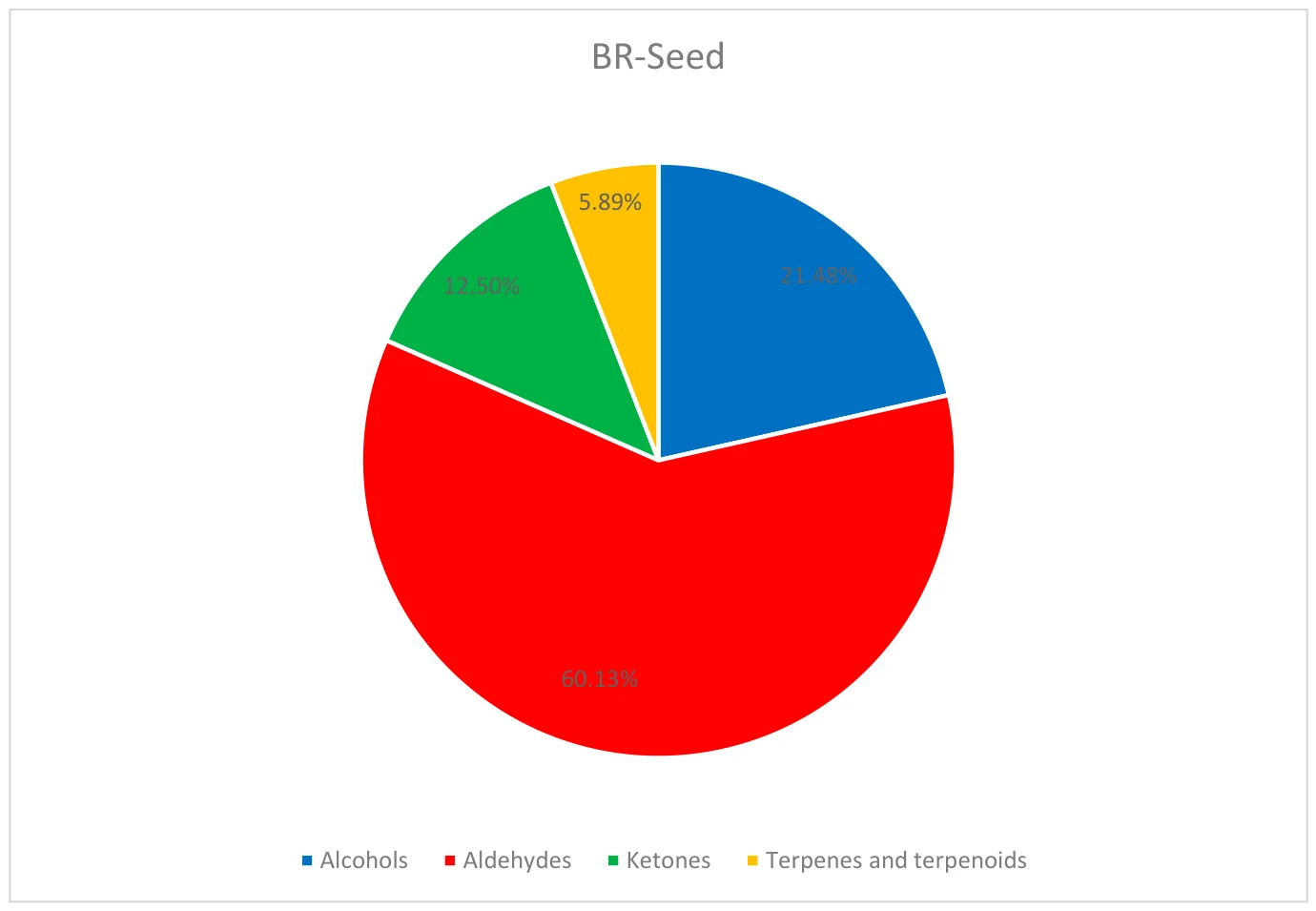
Percentage of relative contribution of compounds from different volatile groups in the volatile profile of rye brome seeds.

**Figure 4:**
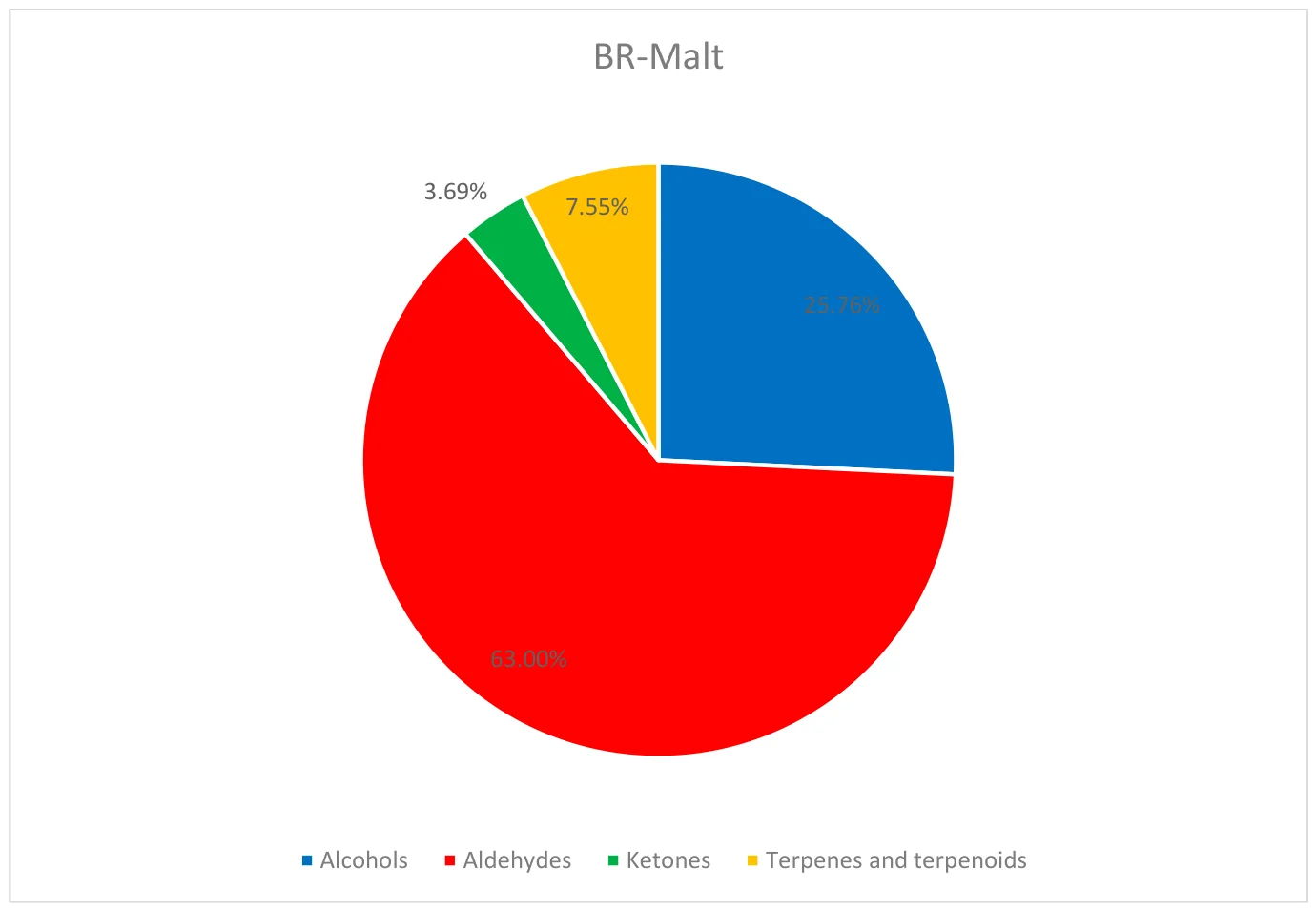
Percentage of relative contribution of compounds from different volatile groups in the total volatile profile of rye brome malts.

**Figure 5:**
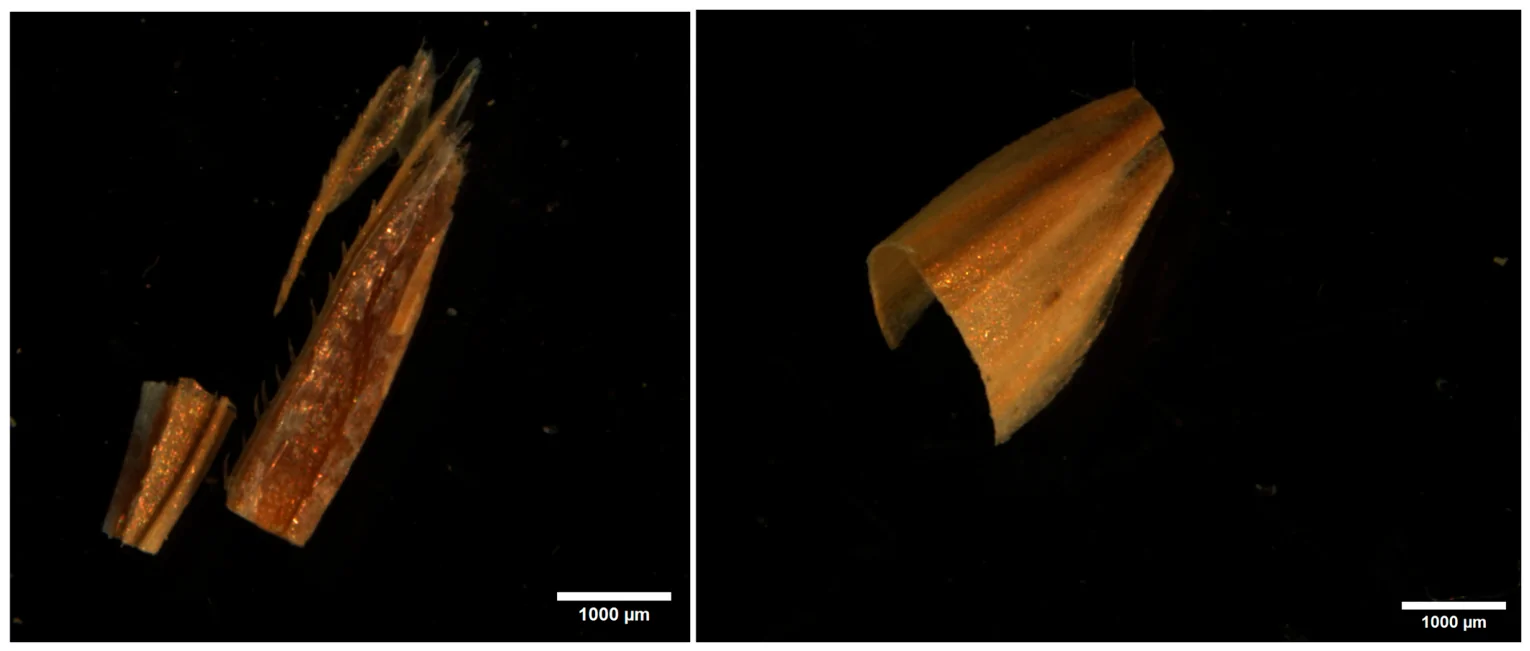

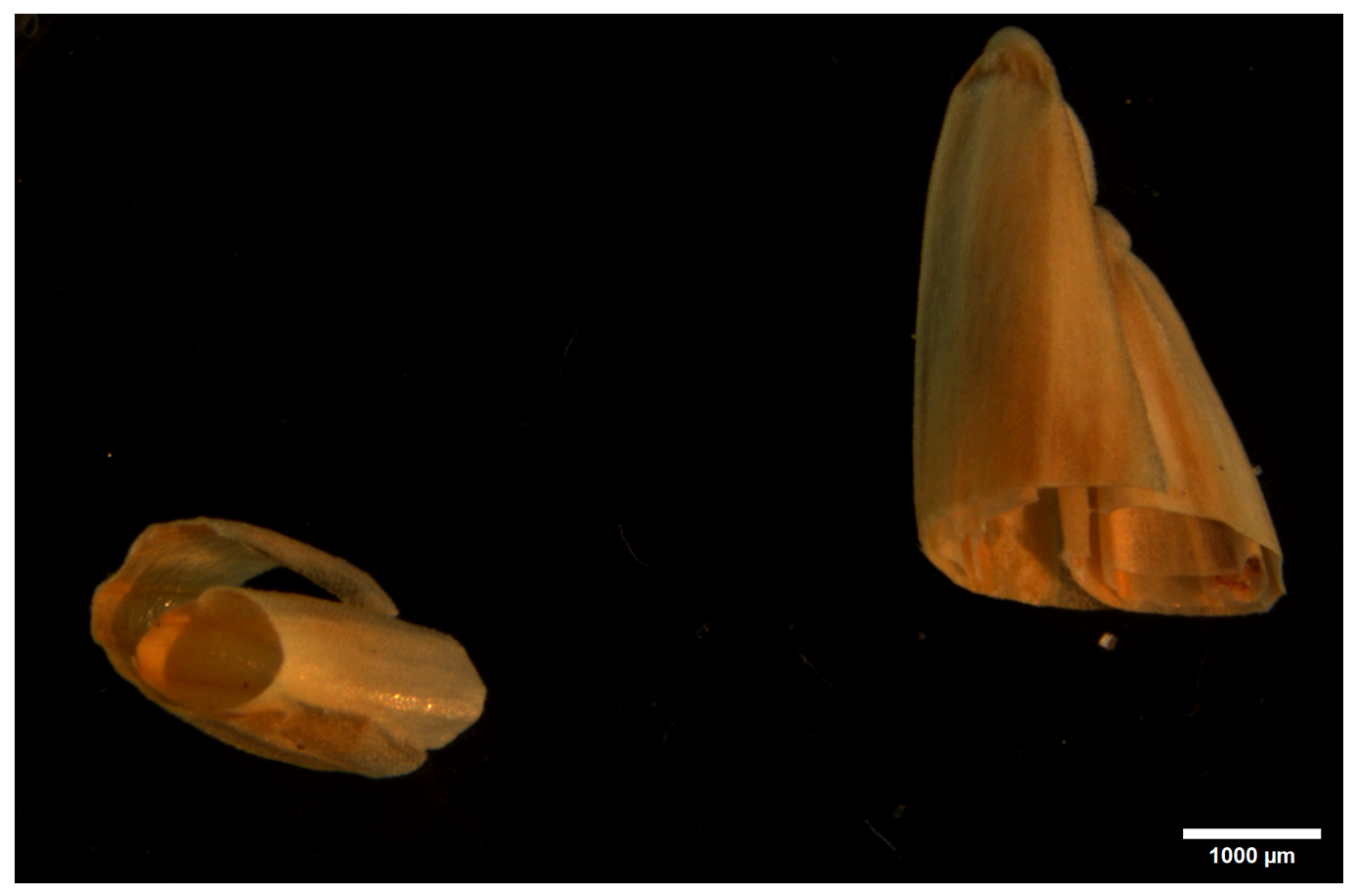
SEM photographs of malted rye brome (seed and husk).

**Table 1:** Perceived aroma, linear retention indices, and methods of identification of volatile compounds in rye brome seeds and malts.

	LRI Exp	LRI Lit	Compound	Reported Odor Descriptor	Method of Identification ^1^	Reference for Aroma
1	851	850	2-Hexenal	grassy, green	LRI, MS, OC	[11]
2	858	865	1-Hexanol	minty, grassy	LRI, MS, OC	[12]
3	890	900	2-Heptanone	cheesy	LRI, MS, OC	[13]
4	904	900	Heptanal	fatty, citrusy, rancid,	LRI, MS, OC	[11]
5	918	913	2-Heptenal	fatty	LRI, MS, OC	[11]
6	924	961	Benzaldehyde	almond	LRI, MS, OC	[14]
7	934	969	1-Heptanol	citrusy, oily	LRI, MS, OC	[15]
8	946	986	1-Octen-3-ol	green, mushroom	LRI, MS, OC	[16]
9	974	1001	Octanal	lemon, green, fat	LRI, MS, OC	[11]
10	992	927	2-Cyclohexen-1-one	rubbery, latex, green	LRI, MS, OC	[17]
11	999	986	2,3-Octanedione	dill, asparagus, cilantro	LRI, MS, OC	[18]
12	1004	1029	1-Hexanol, 2-ethyl-	citrusy	LRI, MS, OC	[19]
13	1015	1037	3-Octen-2-one	earthy, spicy, herbal	LRI, MS, OC	[18]
14	1022	1046	Phenylacetaldehyde	flowery	LRI, MS, OC	[14]
15	1039	1058	2-Octenal	green, nut, fat	LRI, MS, OC	[11]
16	1048	1065	Acetophenone	sweet, pungent, hawthorn	LRI, MS, OC	[18]
17	1055	1068	1-Octanol	fatty, citrus	LRI, MS, OC	[11]
18	1081	1093	3,5-Octadien-2-one	fruity, fatty, mushroom	LRI, MS, OC	[18]
19	1092	n.d.	Ethanone, 1-(2-methyl-1-cyclopenten-1-yl)-	unknown	MS,	
20	1095	1102	Nonanal	floral, citrus	LRI, MS, OC	[20]
21	1141	1142	2,6,6-Trimethyl-2-cyclohexene-1,4-dione	musty, woody, tobacco, leafy	LRI, MS	[21]
22	1189	1189	Ethanol, 2-(2-butoxyethoxy)-	cucumber-like	LRI, MS	[22]
23	1213	1200	Decanal	floral, green	LRI, MS, OC	[17]
24	1225	1221	Ethanol, 2-phenoxy-	cinnamon	LRI, MS	[23]
25	1279	1264	1-Decanol	floral	LRI, MS, OC	[24]
26	1324	1308	Undecanal	soapy, fatty,	LRI, MS, OC	[25]
27	1377	1363	2(3H)-Furanone, dihydro-5-pentyl-	coconut	LRI, MS	[26]
28	1515	1491	Valencene	sweet, fresh, citrusy	LRI, MS, OC	[27]
29	1541	1527	(-)-.alpha.-Panasinsen	unknown	LRI, MS	
30	1575	1609	epi-γ-Eudesmol	sweet, woody, floral	LRI, MS	[28]

^1^ LRI—linear retention indices; MS—mass spectrum; OC—original compound.

**Table 2:** Concentration of volatile compounds in rye brome seeds and rye brome malts.

	Retention Time	Compound	BR-Seed [µg eq./kg DM] ^1^	BR-Malt [µg eq./kg DM]
1	4.261	2-Hexenal	234.53 ± 40.36	53.93 ± 10.65
2	4.535	1-Hexanol	57.97 ± 10.44	16.28 ± 3.21
3	4.889	2-Heptanone	13.13 ± 2.19	n.d.
4	5.121	Heptanal	7.72 ± 1.33	2.09 ± 0.40
5	6.160	2-Heptenal	6.65 ± 1.37	3.95 ± 0.60
6	6.257	Benzaldehyde	25.23 ± 4.42	1.10 ± 0.13
7	6.420	1-Heptanol	9.88 ± 1.31	1.57 ± 0.28
8	6.617	1-Octen-3-ol	13.57 ± 2.33	5.49 ± 0.99
9	7.060	Octanal	6.01 ± 0.80	6.05 ± 1.32
10	7.361	2-Cyclohexen-1-one	5.23 ± 0.95	n.d.
11	7.551	2,3-Octanedione	10.65 ± 1.84	3.26 ± 0.67
12	7.726	1-Hexanol, 2-ethyl-	7.62 ± 1.19	4.34 ± 0.45
13	7.850	3-Octen-2-one	7.75 ± 1.42	1.99 ± 0.38
14	8.125	Phenylacetaldehyde	25.12 ± 4.09	1.14 ± 0.17
15	8.140	2-Octenal	n.d.	2.30 ± 0.32
16	8.266	Acetophenone	3.16 ± 0.50	n.d.
17	8.376	1-Octanol	15.68 ± 2.55	4.02 ± 0.75
18	8.807	3,5-Octadien-2-one	9.70 ± 1.96	1.14 ± 0.46
19	8.984	Ethanone, 1-(2-methyl-1-cyclopenten-1-yl)-	11.30 ± 2.93	n.d.
20	9.033	Nonanal	14.41 ± 2.25	12.72 ± 2.11
21	9.782	2,6,6-Trimethyl-2-cyclohexene-1,4-dione	4.44 ± 0.80	n.d.
22	10.549	Ethanol, 2-(2-butoxyethoxy)-	4.29 ± 0.74	1.69 ± 0.28
23	10.941	Decanal	13.07 ± 1.77	23.52 ± 4.86
24	11.144	Ethanol, 2-phenoxy-	6.90 ± 0.97	5.91 ± 1.01
25	12.015	1-Decanol	3.77 ± 0.46	5.31 ± 1.13
26	12.749	Undecanal	2.23 ± 0.32	2.31 ± 0.30
27	13.615	2(3H)-Furanone, dihydro-5-pentyl-	4.29 ± 0.68	n.d.
28	15.719	Valencene (Naphthalene, 1,2,3,5,6,7,8,8a-octahydro-1,8a-dimethyl-7-(1-methylethenyl)-, [1S-(1.alpha.,7.alpha.,8a.alpha.)]-)	7.20 ± 1.18	2.35 ± 0.32
29	15.851	(-)-.alpha.-Panasinsen	9.64 ± 1.68	3.70 ± 0.72
30	16.275	epi-γ-Eudesmol (2-((2S,4aR)-4a,8-Dimethyl-1,2,3,4,4a,5,6,7-octahydronaphthalen-2-yl)propan-2-ol)	15.98 ± 2.43	7.03 ± 0.92

^1^ Volatile compound levels are reported as estimated relative abundances expressed in µg 2-undecanone equivalents per kilogram of dry matter (µg eq./kg DM); n.d.—compound not detected.

## Data Availability

The original contributions presented in this study are included in the article/Supplementary Material. Further inquiries can be directed to the corresponding author.

## Supplementary Materials

The following supporting information can be downloaded at: https://www.mdpi.com/article/10.3390/foods15193382/s1. Table S1 - Calculation of the chemical classes of volatiles.
